# The NEXT-1 (Next generation pErsonalized tX with mulTi-omics and preclinical model) trial: prospective molecular screening trial of metastatic solid cancer patients, a feasibility analysis

**DOI:** 10.18632/oncotarget.5188

**Published:** 2015-09-09

**Authors:** Seung Tae Kim, Jeeyun Lee, Mineui Hong, Kyunghee Park, Joon Oh Park, Tae Jin Ahn, Se Hoon Park, Young Suk Park, Ho Yeong Lim, Jong-Mu Sun, Jin Seok Ahn, Myung-Ju Ahn, Hee Cheol Kim, Tae Sung Sohn, Dong Il Choi, Jong Ho Cho, Jin Seok Heo, Wooil Kwon, Sang Won Uhm, Hyuk Lee, Byung-Hoon Min, Sung No Hong, Duk Hwan Kim, Sin Ho Jung, Woongyang Park, Kyoung-Mee Kim, Won Ki Kang, Keunchil Park

**Affiliations:** ^1^ Division of Hematology-Oncology, Department of Medicine, Samsung Medical Center, Sungkyunkwan University School of Medicine, Seoul, Korea; ^2^ Innovative Cancer Medicine Institute, Samsung Cancer Center, Samsung Medical Center, Seoul, Korea; ^3^ Department of Pathology & Translational Genomics, Samsung Medical Center, Sungkyunkwan University School of Medicine, Seoul, Korea; ^4^ Samsung Genome Institute, Seoul, Korea; ^5^ Samsung Biological Research Institute, Seoul, Korea; ^6^ Department of Surgery, Samsung Medical Center, Sungkyunkwan University School of Medicine, Seoul, Korea; ^7^ Department of Radiology, Samsung Medical Center, Sungkyunkwan University School of Medicine, Seoul, Korea; ^8^ Department of Thoracic Surgery, Samsung Medical Center, Sungkyunkwan University School of Medicine, Seoul, Korea; ^9^ Division of Pulmonology, Department of Medicine, Samsung Medical Center, Sungkyunkwan University School of Medicine, Seoul, Korea; ^10^ Division of Gastroenterology, Department of Medicine, Samsung Medical Center, Sungkyunkwan University School of Medicine, Seoul, Korea; ^11^ Medical Translational Research Center, Samsung Biological Research Institute, Seoul, Korea; ^12^ Biostatistics and Clinical Epidemiology, Samsung Medical Center, Seoul, Korea

**Keywords:** molecular profiling, genome, ampliseq

## Abstract

We conducted a prospective genomic screening trial with high throughput sequencing and copy number variation (CNV) assay, and immunohistochemistry array in metastatic solid cancer patients. We used Ion AmpliSeq Cancer Hotspot Panel v2 and nCounter Copy Number Variation Assay (21 genes) to identify molecular targets for potential matched therapy. Metastatic solid tumor patients were prospectively consented for molecular profiling tests. The primary outcome for this trial was the feasibility of molecular tests and response rate (matched vs non-matched treatment). Between November 2013 and August 2014, a total of 428 metastatic solid tumor patients were enrolled on to this study. The mutational profiles were obtained for 407 (95.1%) patients. CNV 21-gene assays were successfully performed in 281 (65.7%) of 428 patients. Of the 407 patients with molecular profiling results, 342 (84.0%) patients had one or more aberrations detected. Of the 342 patients, 103 patients were matched to molecularly targeted agents in the context of clinical trials or clinical practice. The response rate was significantly higher in the genome-matched treated group for gastrointestinal/hepatobiliary/rare tumors (matched vs non-matched treatment, 42.6% vs 24.3%, *P* = .009) and lung cancer cohort (matched vs non-matched treatment, 61.2% vs 28.6% < *P* = .001) when compared with the non-matched group. In this trial, we demonstrate that genome-matched treatment based on molecular profiling result in better treatment outcome in terms of response rate.

## INTRODUCTION

With the advent of molecular sequencing era in medical oncology, upfront high-throughput genomic profiling of patient's tumor specimen is now becoming part of clinical trials or clinical research. Furthermore, systematic efforts to characterize the cancer genome constantly add genome alterations to the compilation of potentially actionable genomic alterations [1–7]. Currently, high-throughput genomic sequencing is not conducted routinely in the practice of medical oncology in most cancer centers. There may be several reasons for hindering from rapid application of clinical sequencing in oncology patients: 1) There is a discrepancy between genomic sequencing results and available matched drugs to the sequence in the clinic; 2) the clinical implication of genomic sequencing in each cancer type is not fully understood; 3) currently, unless a master protocol is present for “umbrella” genomic sequencing project which is simultaneously aligned with several matched drugs, the time from target identification through next-generation sequencing (NGS) and the actual application of the drug takes relatively long period of time.

Nevertheless, there have been previous attempts on molecular profiling of patients’ tumors to find potential targets and provide matched trials to the patients. One of the early trials was a pilot study using molecular profiling of 86 patients’ tumors of which 66 of them were provided of matched drugs [8]. One of the largest prospective trials was reported in 2012 which analyzed 1,144 patients with a pool of matched therapy available once genetic aberration is identified. Of 1,144 patients analyzed, 460 (40.2%) had one or more aberration and 175 patients received matched therapy either in clinical trial or as clinical practice [9]. Based on their study, matched targeted therapy was associated with longer time-to-failure compared with their prior systemic therapy in patients with 1 mutation (5.2 vs. 3.1 months, respectively; *P* < .0001).

To address the ongoing challenges in actively incorporating genomics in the diagnosis and treatment of oncology patients, we conducted a prospective NEXT-1 trial with combinatorial approach encompassing somatic mutations, and a panel of CNVs. In this trial, we have utilized 1) the Ion Torrent AmpliSeq Cancer Panel (50 genes) to survey somatic mutations; 2) nanostring copy number variations (CNVs) to survey 21 actionable gene amplifications. The NGS platforms used in this study (AmpliSeq) relies on non-optical detection of hydrogen ions in a semiconductor device [10] and is able to detect 2,855 oncogenic mutations in 50 commonly mutated genes. We and others have shown that AmpliSeq is a highly feasible platform, particularly because it is associated with a low failure rate when using FFPE tissue specimens [11–14].

The primary objectives of this study were to survey the feasibility of genomic profiling in oncology patients and to compare response rate in matched treatment group versus non-matched conventional treatment group in the NEXT-1 trial. Herein, we report the feasibility of the genomic profiling for patients with metastatic cancer who were enrolled in the prospective master protocol study.

## RESULTS

### Patient characteristics and molecular aberrations

Between November 2013 and August 2014, 428 patients enrolled in this study (NEXT-1, *N* = 231; LUNG PERSEQ, *N* = 98). Table 1 provides baseline patient characteristics. All patients were Korean. The most frequent cancer types were gastric cancer (GC; *n* = 133, 31.1%), followed by non-small cell lung cancer (NSCLC; *n* = 99, 23.1%), colorectal cancer (CRC; *n* = 60, 14%), hepatocellular carcinoma (HCC; *n* = 21, 6%), sarcoma (*n* = 25, 5.8%), gastroentro-pancreatic (GEP) neuroendocrine tumor (*n* = 15, 3.5%), melanoma (*n* = 12, 2.9%), cholangiocarcinoma (*n* = 14, 3.3%), and pancreatic adenocarcinoma (*n* = 9, 2.1%).

**Table 1:** Patient Characteristics

Characteristics	No. of Patients (*N* = 428)	%
Sex		
Female	175	40.8
Male	253	59.2
Age. years		
Median	56	
Range	18–82	
ECOG PS		
0–1	415	96.9
≤2	13	3.1
Tumor Types		
Gastric adenocarcinoma	133	31.1
Non-small cell lung cancer	94	22.0
Colorectal adenocarcinoma	60	14.0
Soft tissue sarcoma	25	5.8
Hepatocellular carcinoma	21	4.9
Gastroenteropancreatic neuroendocrine tumor	15	3.5
Cholangiocarcinoma	14	3.3
Melanoma	12	2.8
Pancreatic adenocarcinoma	9	2.1
Small cell lung cancer	5	1.2
Gall bladder cancer	5	1.2
Metastatic carcinoma of unknown origin	4	0.9
GIST*	4	0.9
Thymic carcinoma	4	0.9
Ampulla of Vater cancer	3	0.7
Esophageal squamous carcinoma	3	0.7
Ovarian cancer	3	0.7
Renal cell carcinoma	3	0.7
Non-melanoma skin cancer	3	0.7
Bladder cancer	2	0.5
Uterine/cervix cancer	2	0.5
Adrenocortical carcinoma	1	0.2
Duodenal adenocarcinoma	1	0.2
Glottic cancer	1	0.2
Peritoneal mesothelioma	1	0.2
Metastatic	428	100.0

* Gastrointestinal stromal tumors

**Figure 1:**
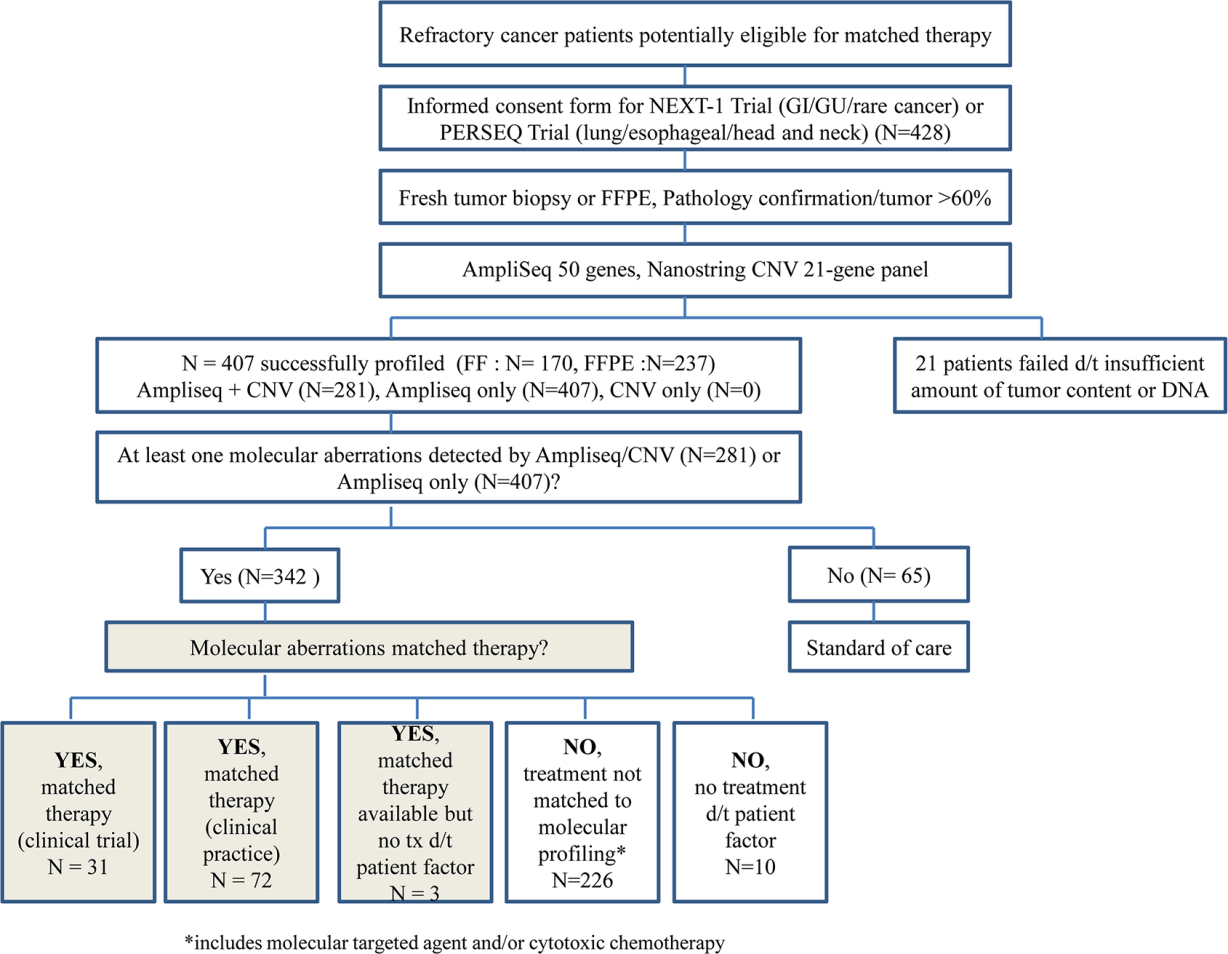
The Study Scheme

**Figure 2:**
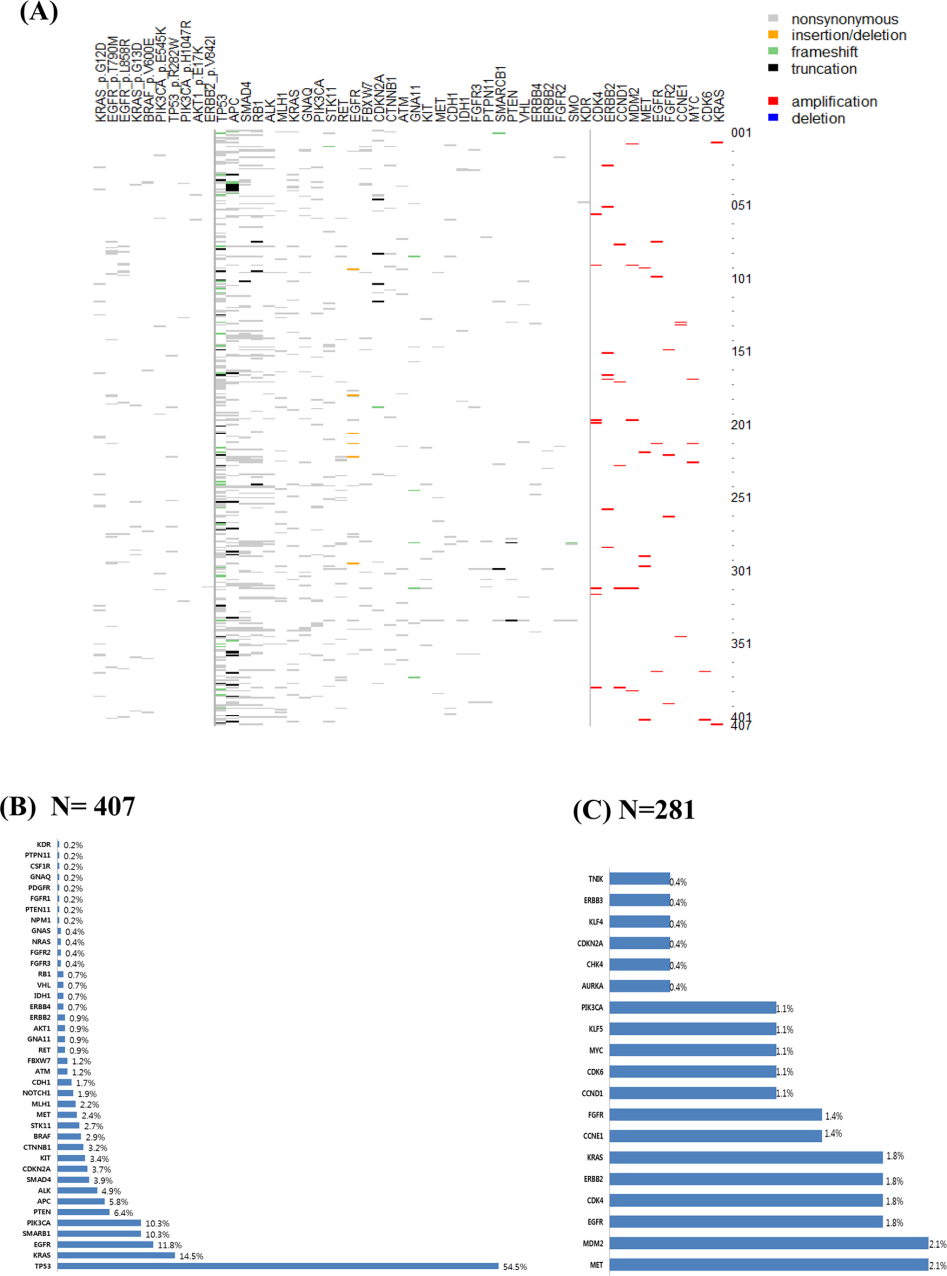
Molecular aberrations in advanced solid tumors **A.** Distribution of somatic mutations and copy number variations in 407 solid cancers **B.** Frequency of somatic mutations (*N* = 407) **C.** Frequency of copy number variations (*N* = 281)

The study scheme is outlined in Fig 1. Of 428 patients, 21 patients (all FFPE) did not enter the genomic analysis due to low tumor cellularity (<10%) (Fig 1). The mutational profiles were obtained in 407 (95.1%) patients using Ampliseq. However, both Ampliseq and CNV 21-gene assays were successfully performed in 281 (65.7%) of 428 patients. 237 (58.2%) specimens were from FFPE tissues, and 170 (41.8%) specimens were from fresh tumor tissues. Most fresh biopsies were obtained from endoscopic/colonoscopic/liver or bronchoscopic biopsies. The median time between biopsy and molecular results was 21 days (range, 17–28 days). Of the 407 patients with cancer panel data available, 342 (84.0%) patients had one or more aberrations detected (Table 2). Mutations were commonly detected in the following genes: *TP53* (54.5%), *KRAS* (14.5%), *EGFR* (11.8%), *SMARCB1* (10.3%), *PIK3CA* (10.3%), *PTEN* (6.4%), and *APC* (5.8%), as shown in Fig 2A and 2B. Most of the genetic aberrations were somatic mutations identified from cancer panel and only 46 out of 281 cases (16.4%) had one or more CNV detected through the CNV 21-gene assay (Table 2, Fig 2B). The most frequently detected amplifications were *MET* (2.1%) and *MDM2* (2.1%), followed by *EGFR* (1.8%), *CDK4* (1.8%), *HER2* (1.8%), *KRAS* (1.8%), *CCNE1* (1.4%), and *FGFR2* (1.4%), as shown in Figure 2C.

**Table 2:** Frequency of Molecular Aberrations

	No. of patients	%
# of patients consented for molecular analysis	428	100
# of specimens quality control passed	407	
No. of aberrations detected for AmpliSeq		
0	65	15.9
1	141	34.6
2	123	30.2
3	51	12.5
≥4	27	6.6
No. of patients with at least one aberrations by AmpliSeq alone	342	84.1
# of specimens available for both Ampliseq and 21-gene copy number variation array	281	
No. of aberrations detected for AmpliSeq and 21-gene copy number variation array*		
0	41	14.6
1	79	28.1
2	81	28.8
3	51	18.1
≥4	29	10.3

* *N* = 407 Ampliseq 2.0 + 21-gene copy number variation array; *N* = 281, Ampliseq 2.0 available

In metastatic GC patients, mutations were frequently detected in *TP53* (43.9%), *PIK3CA* (12.1%), *SMARCB1* (8.1%), *ALK* (7.3%), and *PTEN* (6.5%), as shown in Supplementary Fig 1A and in our previous study.^11^ For NSCLC, *TP53* (90.4%), *EGFR* (48.9%), *SMARCB1* (8.5%), *PTEN* (7.4%), and *KRAS* (7.4%) were frequently mutated (Supplementary Fig 1B). In metastatic CRC patients, the most frequently observed mutations were *TP53* (62.1%), *KRAS* (50.0%), *APC* (31.0%), *PIK3CA* (17.2%), *SMAD4* (13.8%), *SMARCB1* (12.1%), *BRAF* (8.6%), *PTEN* (5.1%), *MET* (5.1%), and *FBXW7* (5.1%) (Supplementary Fig 1C). In HCC, *TP53* (17.6%) and *CTNNB1* (11.7%) were the top two mutations detected with the current platform (Supplementary Fig 1D). In general, the frequencies of somatic mutations detected in this study were comparable to those reported in TCGA. However, the incidence of EGFR mutations (48.6%) was significantly higher in our study cohort when compared with the reported incidence of EGFR mutations in lung cancer (∼10%).^16^

### Molecular profile-based matched treatment

Of the 342 patients with at least one gene aberration, 31 patients were matched to ongoing clinical trial of a molecularly targeted agent (Fig 1). Of 342 patients, 72 patients received matching molecularly targeted agents in the context of clinical practice. Three patients were assigned to matching targeted agents but did not receive the treatment due to deterioration of the disease. 226 patients did not have molecular aberrations that were matched to currently available treatments either in the context of a clinical trial or in clinical practice and were therefore treated with unmatched cytotoxic or other targeted clinical trial drugs. Ten patients did not have matched drugs available and did not receive any treatment after molecular profiling due to medical conditions (i.e., poor performance).

**Figure 3:**
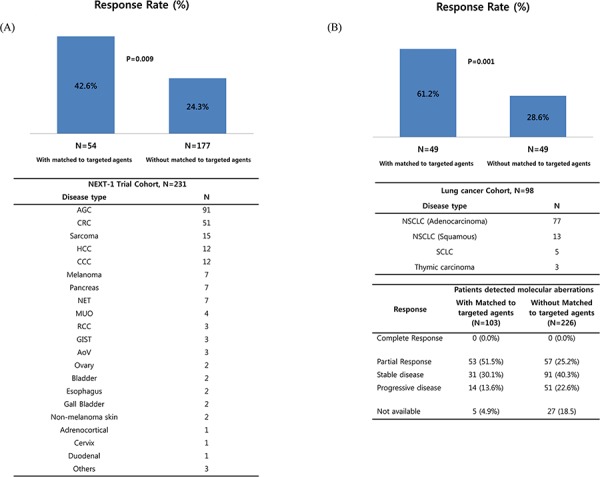
Efficacy data based on molecular profiling. Response rate according to the matched treatment in the NEXT-1 trial cohorts **A.** gastrointestinal/hepatobiliary/rare tumor (*N* = 231) and **B.** lung cancer cohort (*N* = 98).

The primary outcome for this trial was the feasibility of molecular tests and response rate. The response rate for the matched treated group was significantly higher in the genome-matched treated group for gastrointestinal/hepatobiliary/rare tumors (matched vs non-matched, 42.6% vs 24.3%, *P* = .009) and lung cancer cohort (matched vs non-matched, 61.2% vs 28.6% < *P* = .001) (Figs. 3A and 3B).

### Validation of the nanostring CNV 21-gene assay by fluorescence *in situ* hybridization (FISH), immunohistochemistry (IHC), or quantitative real time PCR (qRT-PCR)

**Figure 4:**
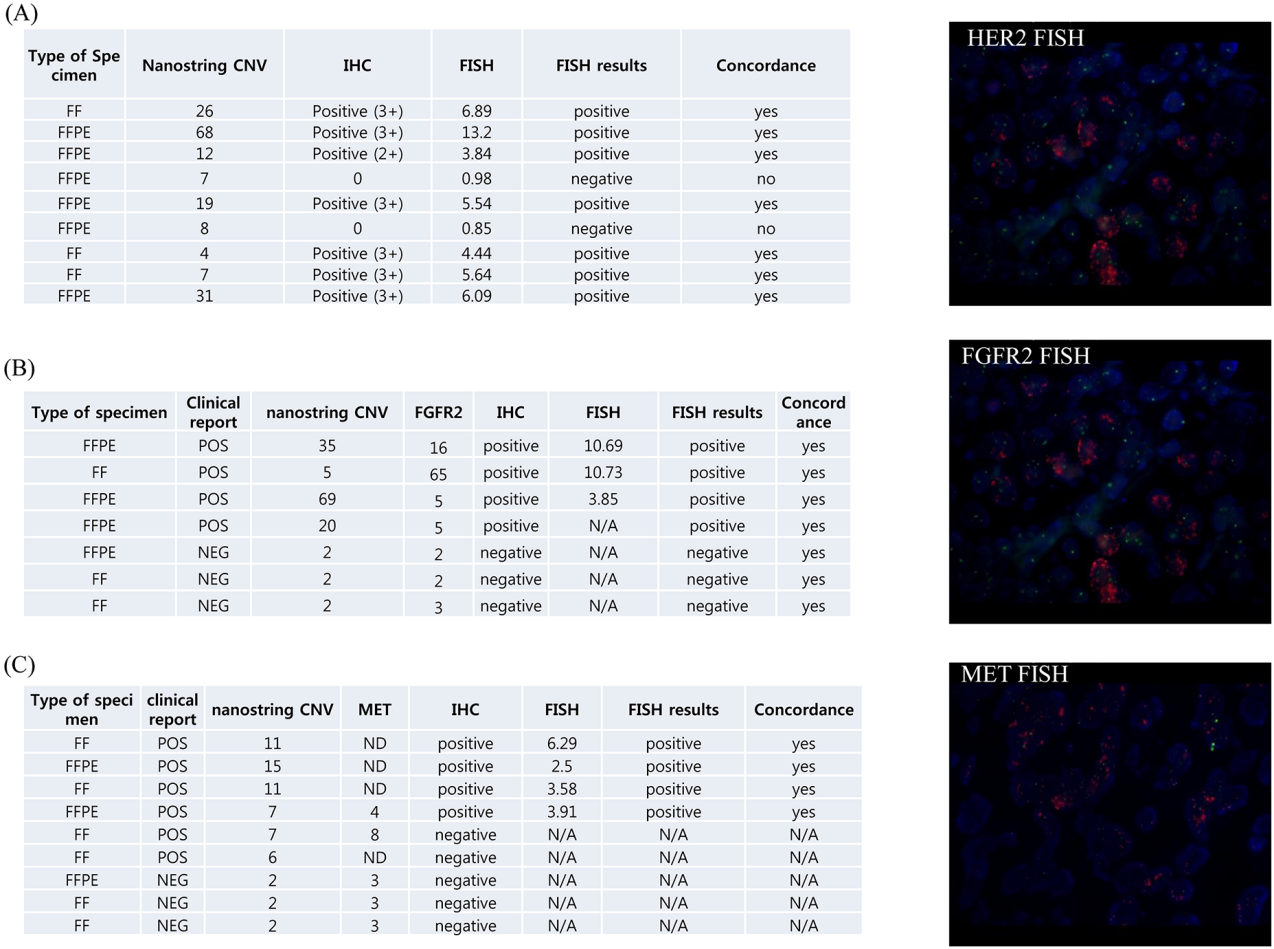
Validation of nanostring CNV 21-gene assay **A.** HER2 amplification **B.** FGFR2 amplification **C.** MET amplification (left panel, validation summary, right panel, representative FISH results). Abbreviations: CNV, copy number variations; IHC, immunohistochemistry; FF, fresh frozen, FFPE, formalin fixed paraffin embedded tissue; concordance between FISH and nanostring CNV.

Next, we validated the results obtained from nanostring 21-gene assays using conventional FISH, IHC, and/or qRT-PCR. In nine selected cases with human epidermal growth factor receptor 2 (*HER2*) positivity in the nanostring assay, seven cases were concordant with conventional *HER2* FISH results (Fig 4A). Two discordant cases exhibited low CNV values in FFPE tissue specimens. For *FGFR2* amplification, the concordance rate was 100% in seven selected cases (Fig 4B). Likewise, the concordance rate for *MET* amplification was 100% in nine selected cases, regardless of the type of tissue specimen (FFPE *versus* fresh; Fig 4C). Validation data for *CCND1*, *CCNE1*, *KRAS*, *PIK3CA*, *CDK4*, and *MDM2* amplifications are provided in the Supplementary Material. In general, the concordance rate between the nanostring 21-gene assay and conventional FISH or IHC was more than 95%, except for *PIK3CA* amplification.

## DISCUSSION

In this study, we validated the clinical feasibility of a prospective molecular screening tool with a combination of cancer panel screening and a nanostring 21-gene CNV assay. The success rate of obtaining the molecular profile was extremely high (95.1%, 407/428) for the cancer panel alone. Given the importance of focal gene amplification, especially in gastrointestinal cancer [5], we designed this screening tool to comprehensively identify actionable mutations and gene amplifications. We were successful in profiling 281 (65.7%) of the enrolled patients to acquire both cancer panel and 21-gene CNV assay data. Importantly, about one-quarter of patients, who underwent molecular screening tests of their tumor samples were matched to a currently ongoing trial or a commercially available drug. The response rate was considerably higher in the genome-matched treated patient cohort when compared with the non-matched treated patient cohort.

Similar trials involving master genomic profiling protocols, which are then matched to available treatments for refractory cancer patients are ongoing. Recently, the interim results of the Molecular Screening for Cancer Treatment Optimization (MOSCATO01) trial were reported in 2013 [17]. From December 2011 to August 2012, 129 heavily pretreated patients (median of three previous treatment lines) consented to participation, and 111 (86%) had dedicated tumor biopsy samples. An actionable target was identified in 52 patients (40%), of whom 25 were treated with a matched targeted therapy. In their report, the PFS ratio was greater than 1.3 among 9 out of 19 evaluable patients (47%), which led to the conclusion that high-throughput molecular analysis was feasible in daily practice. Notably, the percentage of patients undergoing treatment with a matched molecularly targeted agent based on genomic profiling was similar in our study (24.1%) and the MOSCATO01 trial (25%). A list of available targeted drugs is provided in Supplementary Table 3.

The SHIVA trial also reported the feasibility of genomic analysis [18]. In this trial, researchers used an NGS platform similar to the one used in our study (AmpliSeq for cancer panel), but used Cytoscan for CNV analysis and an IHC panel including estrogen receptor (ER), progesterone receptor (PR), and androgen receptor (AR). In the SHIVA trial, the success rate for acquiring mutations was 66%, with a tumor content cut-off of greater than 50%. The success rate for the interim analysis of the SAFIR01 study in breast cancer was 71%, with a 50% threshold for tumor cellularity [19]. Compared with these three large-scale upfront molecular screening trials, the success rate for the NEXT-1/PERSEQ trials was 95% for the AmpliSeq panel, with a 60% cut off for tumor content. While the three above-mentioned trials used mandatory fresh tumor biopsies, only 40% of the specimens were fresh tumors. The success rate for the combined analysis with AmpliSeq and the nanostring CNV panel was 65.7% in this study. In line with other similar molecular screening studies, the accrual rate for the trial was extremely high (428 patients over 10 months).

Despite the high success rate in genomic profiling and the reasonable rate of patients being treated with matched targeted agents, we need to increase the efficiency of the match rate between genomic data and actual treatment. The number of patients who actually received matched therapy in the context of clinical trials according to the genomic profiling was 31 patients, which should be expanded. Currently, we have opened the VIKTORY (targeted agent eValuation in gastric cancer basKeTKORea studY) trial, a GC-specific screening protocol matched to multiple arms of targeted agents as second-line treatment (NCT#02299648) based on molecular profiling. In addition, we are increasing the proportion of fresh tumor biopsies in order to expand the genomic sequencing data and to establish patient-derived cells that are linked to the NEXT-1/VIKTORY and PERSEQ trials. We are now adopting hiseq-based targeted sequencing as an expanded cancer panel, which comprises more than 380 genes as a genomic platform. Based on the feasibility results, the NEXT-1 trial will be modified to interrogate the improved treatment outcome based on genome-matched treatment for refractory cancer patients.

Amplification of oncogenes is a major mechanism for gene overexpression and contributes to tumor development in many cancer types [20]. Traditional methods, such as FISH and array comparative genomic hybridization, are limited by the low resolution of genomic regions and high cost and are labor- and time-consuming. nCounter CNV analysis technology is applicable in FFPE specimens, and the results were extensively validated by IHC and FISH for selected genes, including *MET*, *MDM2*, *CDK4*, *HER2*, and *FGFR2*. The results of nCounter CNV analysis were completely concordant with those of IHC and/or FISH for *MET*, *MDM2*, *FGFR2*, and *CDK4 (supplementary material)*. However, discordance was observed for the HER2 probe. One plausible explanation for this discrepancy may be the poor quality of DNA caused by prolonged cold ischemia time. In all, we identified 46 out of 281 cases (16.4%) with one or more CNV detected through the CNV 21-gene assay. The use of CNV 21-gene assay should be validated further in order to demonstrate a significant increment in identifying matched therapy for metastatic patients. In addition, most of the amplifications that were matched to the actual therapy were gastric cancer. Moreover, we could retrieve CNV results in only 281 patients because high quantity of input DNAs (200ng) required for this assay. Hence, the cost-to benefit ratio to screen all cancer types using nanostring CNV should be carefully determined. Nevertheless, nanostring CNV 21-gene assay facilitated in identifying patients with gene amplification.

We demonstrate that molecular profiling could benefit patients through identifying matched therapy. The inclusion of metastatic cancer patients with only FFPE tissues available will definitely expand on the spectrum of patients who may potentially benefit from molecular screening. We are now on the second phase of the trial with primary endpoint as response rate in matched therapy group based on molecular profiling with expanded list of genes when compared with the non-matched therapy and with expanded list of available matched treatment.

## MATERIALS AND METHODS

### Patients

Patients with metastasis of solid cancers were enrolled onto the NEXT-1 trial [clinicaltrials.gov, NCT#02141152] and LUNG PERSEQ trial [clinicaltrials.gov, NCT#02299622] depending on the cancer types at Samsung Medical Center. The study was approved by the institutional review board of the Samsung Medical Center. All study participants provided written informed consent before study entry. Briefly, patients with metastatic solid cancer who may be potentially enrolled onto the matched therapy were eligible to enter the study. Patients with pathologically confirmed cancer and who had either archived tissue or fresh tissues were eligible for genomic analysis. Patients also consented for *in vitro* establishment of patient-derived cells for research use if tissue was available (this will be reported elsewhere). At the time of genomic analysis, patients were informed of 1) available genome-matched trials, 2) genome-matched treatments in practice, and 3) clinical trials or cytotoxic chemotherapies regardless of available genomic data. The list of available matched therapy at the time of patient accrual is listed in Supplementary Table 1.

### Tumor samples

Tumor areas (>60%) were dissected under microscopy from 4-μm-thick unstained sections by comparison with an H&E stained slide, and genomic DNA was extracted using a Qiagen DNA FFPE Tissue Kit (Qiagen, Hilden, Germany) according to the manufacturer's instructions. After extraction, we measured concentrations and 260/280 and 260/230 nm ratios using a spectrophotometer (ND1000, Nanodrop Technologies, ThermoFisher Scientific, MA, USA). Each sample was then quantified with a Qubit fluorometer (Life Technologies, Carlsbad, CA, USA). Samples of genomic DNA with more than 10 ng measured by the Qubit fluorometer were subjected to AmpliSeq library preparation. To identify actionable CNVs, we used a 21-gene nCounter CNV assay, as previously described [11, 15]. An AmpliSeq cancer panel v2 was examined.

### Ion ampliseq cancer panel v2

We used the Ion AmpliSeq Cancer Panel v2 (Ion Torrent) to detect frequent somatic mutations. Detailed methods were published previously [11]. Briefly, this assay examines 2,855 mutations in 50 commonly mutated oncogenes and tumor-suppressor genes. We used IonTorrent variant caller (version 4.0) for automated data analysis.

### nCounter 21-gene CNV assay

We custom designed a 21-gene CNV assay as outlined in a previous study [11]. We selected 21 genes based on available targeted agents at the time of study design. The 21 genes included *AURAKA, CCND1, CCNE1, CDK4, CDK6, CDNK1A, CDNK2A, EGFR, ERBB2, ERBB3, FGFR1, FGFR2, IGFR1R, KLF5, KRAS, MDM2, MET, MITF, MYC, PIK3CA, and TNIK*. For detection of CNVs, nCounter Copy Number Variation CodeSets were used with 200 ng purified genomic DNA. DNA was fragmented via AluI digestion and denatured at 95°C. Fragmented DNA was hybridized with the codeset of 21 genes in the nCounter Cancer CN Assay Kit (Nanostring Technologies) for 18 h at 65°C and processed according to the manufacturer's instructions (Supplementary Table 2) [16]. The nCounter Digital Analyzer counted and tabulated the signals of reporter probes. Quantified data were analyzed using NanoString's nSolver Analysis Software. For normalization of the results, human universal genomic DNA (PR-G3041, Promega) was used. To determine cut off value, we validated nCounter results using conventional FISH, IHC, and/or qRT-PCR and the cut off value was ≥3 in cases using DNAs extracted from fresh tissue and ≥v5 in cases using DNAs from formalin-fixed paraffin embedded tissue samples. In cases with poor DNA quality, the CNV panel resulted in high value in almost all genes and caution in interpretation was needed. Tumor tissues from 281 patients were available for CNV analysis. In cases with gene amplification identified through the nanostring CNV assay, confirmation using FISH and real-time qRT-PCR was conducted (Supplementary Table 3). Detailed protocols for qRT-PCR, IHC, and FISH are provided in the previous study [10].

### Analytical methods

We excluded all synonymous changes after an automated mutation-calling algorithm was used to detect supposed mutations. We used cutoff values of more than 6% variant frequency and more than X100 coverage to detect true mutational changes in accordance with previous studies and our own experience. We filtered out single-nucleotide polymorphisms after manual review of each polymorphism in the Catalogue of Somatic Mutations in Cancer (COSMIC, http://cancer.sanger.ac.uk/cancergenome/projects/cosmic). In order to reduce the potential false-positive calls, the following criteria were used to generate final variant calling. The filtering of the variants was performed by analyzing the coverage (>100 ×), quality score (>30), and variant frequency of each sample (>1%). We also discarded Korean-specific germline variants, such as rs1042522 in TP53 and rs1870377 in KDR. Only functional mutations were included in further analysis. We used the MyCancerGenome database (http://www.mycancergenome.org/) to address clinically actionable mutations. A heat map was generated using R version 3.0.2 with the gplots package. For well-known genes mutated in each cancer type, a manual review of automated calling results was performed to catch deleterious mutations using the Integrative Genomic Viewer (Broad Institute) with slightly low variant frequency. Patients’ characteristics were analyzed using descriptive statistics. Categorical data were described using contingency tables, including counts and percentages.

## SUPPLEMENTARY TABLES AND FIGURE


