# Supplementary material for: The NEXT-1 (Next generation pErsonalized tX with mulTi-omics and preclinical model) trial: prospective molecular screening trial of metastatic solid cancer patients, a feasibility analysis
Source: Oncotarget. 2015 Sep 9;6(32):33358–68. doi: 10.18632/oncotarget.5188 (PMC4741771; doi:10.18632/oncotarget.5188)
Supplement: Supplementary file 1 [file oncotarget-06-33358-s001.pdf]

SUPPLEMENTARY TABLES AND FIGURE

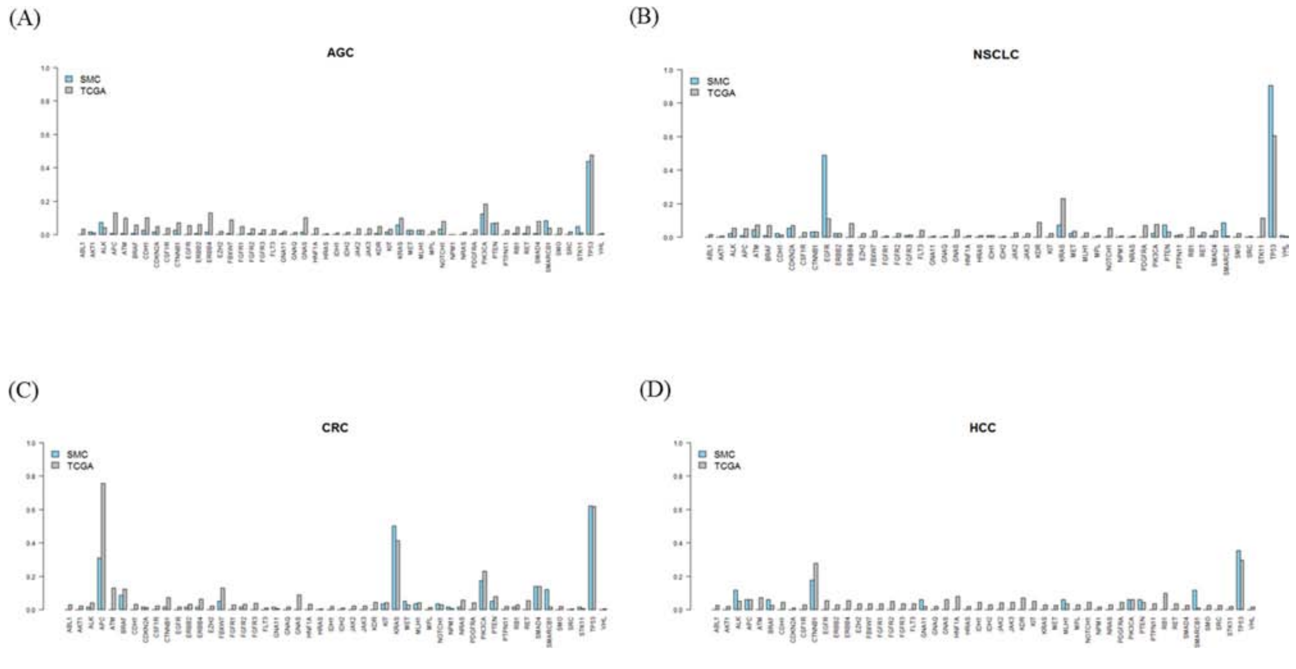

Supplementary Figure S1: Molecular aberrations in specific tumor types as compared with those of TCGA data. A. Somatic mutations in gastric cancer (n = 123) **B.** non-small cell lung cancer (n = 94) **C.** colon cancer (N = 58) **D.** hepatocellular carcinoma (n = 17).

Supplementary Table S1: Molecular aberrations matched to targeted agents

Molecular aberrations matched to targeted agents in clinical trials, $N = 31$			
**Molecular Target Identified**	**Matched Therapy**	**Disease type**	N
ALK (IHC)	Crizotinib	Non-small cell lung cancer	1
BRAF mutation	Vemurafenib	MelanomaMetastatic carcinoma of unknown origin	3
HER2 amplification	Pertuzumab	Gastric adenocarcinoma	4
KRAS wild-type	Panitumumab	Colorectal adenocarcinoma	1
EGFR mutation	Afatinib	Non-small cell lung cancer	3
HER2 amplification	Lapatinib	Gastric adenocarcinoma	1
MET amplification	Anti-MET targeted agent	Gastric adenocarcinoma	1
EGFR T790M	Anti-T790M targeted agent[a]	Non-small cell lung cancer	7
EGFR T790M	Anti-T790M targeted agent[b]	Non-small cell lung cancer	3
EGFR mutation	Anti-EGFR targeted agent[c]	Non-small cell lung cancer	3
EGFR mutation	Anti-EGFR targeted agent[d]	Non-small cell lung cancer	3
FGFR2 amplification	Anti-FGFR targeted agent	Gastric adenocarcinoma	1
**Molecular aberrations matched to targeted agents in clinical practice, $N = 72$**			
**Molecular Target Identified**	**Matched Therapy**	**Disease type**	N
EGFR mutation	Erlotinib	Non-small cell lung cancerPancreatic adenocarcinoma	13
EGFR mutation/ amplification	Gefitinib	Non-small cell lung cancerMetastatic carcinoma of unknown origin	19
HER2 amplification	Trastuzumab	Gastric adenocarcinoma	15
KRAS mutation	Cetuximab	Colorectal adenocarcinoma	14
KIT amplification/mutation	Imatinib	GIST*, Soft tissue sarcoma	4
PIK3CA mutation	Everolimus	Renal cell carcinoma	2
	Pazopanib	Soft tissue sarcoma	4
BRAF mutation	Sorafenib	Hepatocellular carcinoma	1

*Gastrointestinal stromal tumors

Supplementary Table S2: List of customized NanoString CNV codeset and matched TaqMan Copy Number Assay IDs

NanoString CNV codeset			matched qPCR assay
Gene Name	Genome Build	Genomic Coordinates	TaqMan Copy Number Assay ID
CCND1	Hg18	chr11:69166461–69166560	Hs03772544_cn (CCND1_1)
CCND1	Hg18	chr11:69170198–69170280	Hs03776261_cn (CCND1_2)
CCND1	Hg18	chr11:69173802–69173877	Hs02666923_cn (CCND1_3)
CCNE1	Hg18	chr19:34996365–34996462	Hs00772084_cn (CCNE1_1)
CCNE1	Hg18	chr19:35000303–35000396	Hs00452646_cn (CCNE1_2)
CCNE1	Hg18	chr19:35005181–35005262	Hs01813172_cn (CCNE1_3)
KRAS	Hg18	chr12:25261832–25261919	Hs06927012_cn (KRAS_1)
KRAS	Hg18	chr12:25275152–25275241	Hs06962121_cn (KRAS_2)
KRAS	Hg18	chr12:25285835–25285922	Hs06973152_cn (KRAS_3)
PIK3CA	Hg18	chr3:180415123–180415222	Hs00725627_cn (PIK3CA_1)
PIK3CA	Hg18	chr3:180423950–180424049	Hs00185640_cn (PIK3CA_2)
PIK3CA	Hg18	chr3:180432391–180432478	Hs02708380_cn (PIK3CA_3)
MET	Hg18	chr7:116136002–116136089	Hs05008121_cn (MET_1)
MET	Hg18	chr7:116160904–116161003	Hs04985499_cn (MET_2)
MET	Hg18	chr7:116214262–116214361	Hs05020704_cn (MET_3)
FGFR1	Hg18	chr8:38393783–38393874	Hs02702320_cn (FGFR1_1)
FGFR1	Hg18	chr8:38413246–38413333	Hs06184858_cn (FGFR1_2)
FGFR1	Hg18	chr8:38432656–38432745	Hs05051339_cn (FGFR1_3)
FGFR2	Hg18	chr10:123248163–123248262	Hs05182482_cn (FGFR2_1)
FGFR2	Hg18	chr10:123290793–123290892	Hs05176095_cn (FGFR2_2)
FGFR2	Hg18	chr10:123325834–123325907	Hs05114893_cn (FGFR2_3)
MDM2	Hg18	chr12:67498111–67498194	Hs03058884_cn (MDM2_1)
MDM2	Hg18	chr12:67504615–67504714	Hs03013618_cn (MDM2_2)
MDM2	Hg18	chr12:67518205–67518304	Hs06362713_cn (MDM2_3)
AURKA	Hg18	chr20:54380758–54380852	
AURKA	Hg18	chr20:54387730–54387829	
AURKA	Hg18	chr20:54395073–54395172	
CDK4	Hg18	chr12:56428625–56428714	
CDK4	Hg18	chr12:56430637–56430731	
CDK4	Hg18	chr12:56430858–56430945	
CDK6	Hg18	chr7:92102617–92102714	
CDK6	Hg18	chr7:92192438–92192525	
CDK6	Hg18	chr7:92279486–92279585	

(*Continued*)

NanoString CNV codeset			matched qPCR assay
Gene Name	Genome Build	Genomic Coordinates	TaqMan Copy Number Assay ID
CDKN1A	Hg18	chr6:36760296–36760371	
CDKN1A	Hg18	chr6:36760902–36760995	
CDKN1A	Hg18	chr6:36761312–36761391	
CDKN2A	Hg18	chr9:21963369–21963468	
CDKN2A	Hg18	chr9:21964186–21964285	
CDKN2A	Hg18	chr9:21965829–21965928	
EGFR	Hg18	chr7:55072102–55072181	
EGFR	Hg18	chr7:55130363–55130434	
EGFR	Hg18	chr7:55188580–55188673	
ERBB2	Hg18	chr17:35112859–35112934	
ERBB2	Hg18	chr17:35124948–35125029	
ERBB2	Hg18	chr17:35135268–35135347	
ERBB3	Hg18	chr12:54764300–54764394	
ERBB3	Hg18	chr12:54771042–54771141	
ERBB3	Hg18	chr12:54778487–54778586	
IGF1R	Hg18	chr15:97040030–97040129	
IGF1R	Hg18	chr15:97164717–97164804	
IGF1R	Hg18	chr15:97284983–97285082	
KLF5	Hg18	chr13:72534198–72534277	
KLF5	Hg18	chr13:72539794–72539893	
KLF5	Hg18	chr13:72546812–72546911	
MITF	Hg18	chr3:69914121–69914220	
MITF	Hg18	chr3:69997161–69997260	
MITF	Hg18	chr3:70077697–70077786	
MYC	Hg18	chr8:128818714–128818790	
MYC	Hg18	chr8:128820773–128820868	
MYC	Hg18	chr8:128821228–128821321	
TNIK	Hg18	chr3:172563186–172563285	
TNIK	Hg18	chr3:172603694–172603790	
TNIK	Hg18	chr3:172643608–172643707	

Supplementary Table S3: Validation of nanostring CNV assay using quantitative PCR, FISH, IHC

Gene	Type of cancer	Type of Specimen	Clinical Report	Nanostring CNV	matched qPCR assay			Results ofIHC	Copy number by FISH
**CCND1 amp**					CCND1_1 CN Predicted	CCND1_2 CN Predicted	CCND1_3 CN Predicted		
	melanoma, colon	FF	POS	15	15	15	13		
	adenocarcinoma, colon	FF	POS	7	4	7	5		
	adenocarcinoma, colon	FF	NEG	2	2	3	2		
	adenocarcinoma, rectum	FF	NEG	2	3	3	3		
	melanoma, pleura	FFPE	POS	8	2	7	3		
	melanoma, maxilla	FFPE	POS	20	22	31	21		
	adenocarcinoma, stomach	FFPE	POS	11	5	6	5		
	adenocarcinoma, stomach	FFPE	NEG	2	3	3	3		
	adenocarcinoma, stomach	FFPE	NEG	2	1	3	1		
**CCNE1 amp**					CCNE1_1 CN Predicted	CCND1_2 CN Predicted	CCND1_ 3CN Predicted		
	neuroendocrine carcinoma, lymph node	FFPE	POS	19	60	ND	33		
	adenocarcinoma, stomach	FFPE	POS	6	20	6	6		
	adenocarcinoma, stomach	FFPE	POS	10	35	11	9		
	adenocarcinoma, stomach	FFPE	POS	18	53	1	20		
	adenocarcinoma, stomach	FFPE	NEG	2	8	3	2		
	adenocarcinoma, rectum	FF	NEG	2	3	3	3		
**KRAS amp**					KRAS_1 CN Predicted	KRAS_2 CN Predicted	KRAS_3 CN Predicted		
	adenocarcinoma, colon	FF	POS	21	28	28	32		

(*Continued*)

Gene	Type of cancer	Type of Specimen	Clinical Report	Nanostring CNV	matched qPCR assay			Results ofIHC	Copy number by FISH
	adenocarcinoma, rectum	FF	POS	17	22	23	25		
	adenocarcinoma, rectum	FF	POS	6	5	4	4		
	adenocarcinoma, stomach	FFPE	POS	16	9	10	10		
	adenocarcinoma, stomach	FFPE	POS	11	0	1	1		
	adenocarcinoma, colon	FF	NEG	2	2	2	2		
	adenocarcinoma, stomach	FFPE	NEG	3	1	2	1		
**PIK3CA amp**					PIK3CA_1 CN Predicted	PIK3CA_2 CN Predicted	PIK3CA_3 CN Predicted		
	adenocarcinoma, stomach	FFPE	POS	13	0	0	0		
	urothelial carcinoma, bladder	FF	POS	4	5	6	5		
	adenocarcinoma, stomach	FFPE	NEG	2	3	4	2		
	adenocarcinoma, stomach	FFPE	NEG	2	2	2	1		
	adenocarcinoma, rectum	FF	NEG	2	3	2	2		
	adenocarcinoma, colon	FF	NEG	2	3	3	3		
**CDK4 amp**									
	adenocarcinoma, stomach	FF	POS	29				positive	N/A
	melanoma, pleura	FFPE	POS	59				positive	3.72
	inflammatory malignant fibrous histiocytoma, soft tissue	FFPE	POS	10				positive	3.45
	melanoma, maxilla	FFPE	POS	66				N/A	N/A
	undifferentiated pleomorphic sarcoma, lung	FFPE	POS	6				positive	2.75

(*Continued*)

Gene	Type of cancer	Type of Specimen	Clinical Report	Nanostring CNV	matched qPCR assay			Results ofIHC	Copy number by FISH
**MDM2 amp**					MDM2_1 CN Predicted	MDM2_2 CN Predicted	MDM2_3 CN Predicted		
	spindle cell sarcoma, lung	FF	POS	21	19	18	14	positive	N/A
	melanoma, maxilla	FFPE	POS	28	60	46	45	positive	N/A
	adenocarcinoma, stomach	FFPE	POS	6	5	4	7	positive	5.11
	alveolar rhabdomyosarcoma, lymph node	FFPE	POS	17	0	0	7		
	adenocarcinoma, stomach	FFPE	NEG	2	2	2	3		
	adenocarcinoma, stomach	FFPE	NEG	2	1	1	2		
	adenocarcinoma, rectum	FFPE	NEG	1	2	2	2		
	appendiceal cancer, appendix	FF	NEG	1	2	2	2		
